# Supplementary material for: Barriers and promoters to adapting research findings to clinical care in hereditary angioedema in the United States: A qualitative study
Source: PLoS One. 2025 Oct 9;20(10):e0329585. doi: 10.1371/journal.pone.0329585 (PMC12510502; doi:10.1371/journal.pone.0329585)
Supplement: Attachment — Submitted filename: HAE-qualitative_PLOS_Response to Reviewers_Resubmit_031325.docx [file pone.0329585.s001.docx]

**Response to Reviewers**

**March 13, 2025**

Dear PLOS ONE Editors and Staff:

Thank you for reviewing our previously submitted manuscript, PONE-D-25-11285, entitled "Barriers and promoters to adapting research findings to clinical care in hereditary angioedema: a qualitative study."

We appreciate the invitation to **resubmit** the CORRECTED and REVISED manuscript for your consideration.

Herein we respond point-by-point to the comments in the Rejection Notice dated March 10, 2025.

**The Rejection Notice stated:**

"We recognize that you obtained ethical approval from your local ethics committee after you began the study: your ethics exemption document is dated December 13, 2023, while participants were recruited beginning in August 2, 2023. For human subjects research, PLOS ONE requires that authors obtain ethics committee approval before beginning the study, and we do not accept retrospective ethical approval."

**Our Response:**

CORRECTION

The recruitment process for this study started on JANUARY 9, 2024.

It did not begin on August 2, 2023, as stated in the previously submitted Human Participants Research Checklist dated March 4, 2025.

CLARIFICATION

The CME activity, but not subject recruitment, started on August 2, 2023. Approval from the Solutions IRB/ethics committee was obtained on December 13, 2023. Recruitment began on January 9, 2024, after IRB approval.

The first participant interview was carried out on January 16, 2024, as stated in the Results section. The last interview occurred on June 27, 204. Recruitment ended on June 27, 2024.

REASON FOR CORRECTION

The statement listing August 2, 2023, on the initial Human Subjects Participants Research Checklist dated March 4, 2025, was made in error, due to a clerical mistake and misunderstanding on the part of the corresponding author.

The authors regret this error and attest that they have directly verified via study records review that recruitment started on JANUARY 9, 2024. No individuals were approached in any manner to participate in this study before JANUARY 9, 2024.

A corrected Human Subjects Participants Research Checklist was uploaded to the Submission File today, along with the certificate of approval from the Solutions IRB/ethic committee dated December 13, 2023.

Pertinent portions of the Methods section have therefore been CORRECTED to read as follows:

“The continuing medical education (CME) activity *Case Studies in Hereditary Angioedema: Moving Beyond Crisis to Long-Term Prevention* was available for 12 months on the educational platform myCME.com starting on August 2, 2023. The activity was intended for allergists, immunologists, primary care physicians (PCPs), and advanced practice providers involved in those specialties. The activity was certified for 1.5 *AMA PRA Category 1 Credits*^TM^ by Partners for Advancing Clinical Education (PACE). The faculty person for the activity was one of the authors (WL).

The study was verified to be exempt according to 45 CFR 44.101(b)(2) by an independent review board (Solutions IRB) on December 13, 2023. The recruitment process for this study started on January 9, 2024, and ended on June 27, 2024. Beginning on January 9, 2024, learners who had completed the CME activity were asked via email if they wanted to be interviewed about the translation of research results into patient care related to HAE. Learners who were interested in participating and were allergy/immunology clinicians were invited by email to be interviewed. Additional allergy/immunology physicians were invited from a geographically diverse range across the United States, as identified through healthcare provider directories.

The risks and benefits of participation in this study were explained and presented in writing to all potential interviewees prior to the interview. All participants provided verbal consent before starting the interview. One investigator (EL; physician) obtained and documented the verbal consent and conducted the interview online (audio only). Audio recordings were transcribed verbatim. Participant data and personal information were deidentified. Data were read, analyzed, and coded by one investigator (DR) with coding reviewed by all investigators. Interviews and notes were coded using Dedoose, a web-based application for qualitative analysis and mixed methods research.^8”^

We hope that PLOS ONE will reconsider its decision, based on the correction and revision above. If more information is required, we will be pleased to provide it to you expeditiously.

Thank you,

Esther Langmack, MD, FCCP, CHCP

Corresponding Author

elangmack@gmail.com
