# Supplementary material for: Barriers and promoters to adapting research findings to clinical care in hereditary angioedema in the United States: A qualitative study
Source: PLoS One. 2025 Oct 9;20(10):e0329585. doi: 10.1371/journal.pone.0329585 (PMC12510502; doi:10.1371/journal.pone.0329585)
Supplement: Attachment — Submitted filename: Response to Reviewers_052425.docx [file pone.0329585.s002.docx]

**RESPONSE TO REVIEWERS**

**Re: PONE-D-25-13647**

**May 24, 2025**

**To:**	Miquel Vall-Illosera Camps

Senior Staff Editor

PLOS One

Thank you for your kind consideration of our manuscript (PONE-D-25-13647).

Please be aware that this manuscript now has a new title: *Barriers and promoters to adapting research findings to clinical care in hereditary angioedema in the U.S.: a qualitative study*. The title was changed in response to a Reviewer #1 comment.

We appreciate the editors’ and the reviewer’s constructive suggestions and have made the following changes in response.

1. We modified the manuscript to meet PLOS One’s style requirements. A detailed record of all changes can be viewed in the uploaded Revised Manuscript with Track Changes.
2. We uploaded our de-identified, anonymized data to Harvard Dataverse, a repository recommended by PLOS One. The DOI for our dataset is: <https://doi.org/10.7910/DVN/ZBIFEF>
   a. The dataset DOI is mentioned and cited in the Methods section of the paper (Manuscript, Line 109)
   b. The reference list contains a citation for the dataset (Reference 9, Manuscript, Line 571)
   c. The Data Availability statement in the submission form now reflects the new dataset repository
3. At the request of the Journal editors, we have added the statement “This does not alter our adherence to PLOS One policies on sharing data and materials” to the Competing Interests statement in our cover letter. Please see the Cover Letter submitted today for the revised Competing Interests statement.
4. We responded to the Reviewer Comments to the Author, as described below.

**Comments to the Author**

***1. Is the manuscript technically sound, and do the data support the conclusions?***

*The manuscript must describe a technically sound piece of scientific research with data that supports the conclusions. Experiments must have been conducted rigorously, with appropriate controls, replication, and sample sizes. The conclusions must be drawn appropriately based on the data presented.*

*Reviewer #1: Yes*

***2. Has the statistical analysis been performed appropriately and rigorously?***

*Reviewer #1: N/A*

***3. Have the authors made all data underlying the findings in their manuscript fully available?***

*The* [*PLOS Data policy*](http://track.editorialmanager.com/CL0/http:%2F%2Fwww.plosone.org%2Fstatic%2Fpolicies.action%23sharing/1/010f0196b3e65d27-8246177e-753f-40cd-9747-73b3b15e573e-000000/vC1j2wwZ8UKrskLbQ4lefEkc_IpDyzrtTBUGOyoDIFI=210) *requires authors to make all data underlying the findings described in their manuscript fully available without restriction, with rare exception (please refer to the Data Availability Statement in the manuscript PDF file). The data should be provided as part of the manuscript or its supporting information, or deposited to a public repository. For example, in addition to summary statistics, the data points behind means, medians and variance measures should be available. If there are restrictions on publicly sharing data—e.g. participant privacy or use of data from a third party—those must be specified.*

**AUTHOR RESPONSE:** We have uploaded our de-identified, anonymized data to Harvard Dataverse, a repository recommended by PLOS One. The DOI for our dataset is: <https://doi.org/10.7910/DVN/ZBIFEF> The dataset has been cited in the Methods section of the manuscript (Manuscript, Line 108-109), and a citation for the dataset repository appears in the reference list (Manuscript, Reference 9, Line 571).

***4. Is the manuscript presented in an intelligible fashion and written in standard English?***
 *PLOS ONE does not copyedit accepted manuscripts, so the language in submitted articles must be clear, correct, and unambiguous. Any typographical or grammatical errors should be corrected at revision, so please note any specific errors here.*

*Reviewer #1: Yes*

***5. Review Comments to the Author***

***Reviewer #1****: In this manuscript by Langmark et al., significant issues related to the translation in clinical practice of research topics related to hereditary angioedema (HAE) management were analyzed by an interview approach with allergy/immunologists practicing in the USA. The scope of the work was to highlight barriers and promoters of such translations. Major barriers that emerged from the interviews were 1) insurance prior authorization for medications, including drugs for long-term prophylaxis of HAE attacks, 2) laboratory testing difficulties for HAE diagnosis, 3) time constraints for physicians and patient care; while promoters of translations were: 1) availability of medications samples, 2) route/frequency of administration (for LTP), and 3) shared-decision making.
The study, presented using an overall narrative approach, adds some relevant information on the clinical management of HAE patients. Notwithstanding, it applies to the specific USA healthcare setting, whereby, for example, the issue of insurance prior authorization for medications does not apply in Countries based on a public healthcare system.
Based on the mean experience of interviewed physicians, it is clear that they are not HAE specialists. HAE is a true rare disease and is handled in many Countries (especially in Europe) within a network of rare disease centers with HAE specialists who take care of patients.
I would therefore suggest to the AA. to recall in the title that the study was carried on in the USA.
Also, I would suggest making a comment in the discussion about the specific setting of this study, whereby general allergy specialists or general practitioners participated in the study, therefore, there could be a different scenario if the study had been carried out with HAE specialists.*

**AUTHOR RESPONSE:** We agree that clinical practices and challenges associated with HAE care vary around the world and that it is necessary to stipulate that our study involved health care providers located only in the United States. Accordingly, we have made the following changes:

a. As suggested by Reviewer #1, we changed the title from “*Barriers and promoters to adapting research findings to clinical care in hereditary angioedema: a qualitative study*” to “*Barriers and promoters to adapting research findings to clinical care in hereditary angioedema in the U.S.: a qualitative study*” (Manuscript, Line 1)
b. As suggested by Reviewer #1, we specifically acknowledge that U.S. clinical practice characteristics and challenges may differ from those elsewhere in the world in the Discussion (Manuscript, Line 525-530), which has been changed to read as follows (changes are underlined):

Potential limitations to this study include the fact that it enrolled a non-random sample of US allergy/immunology health professionals, the majority of whom were general allergy practitioners in group practices, whose perspectives may potentially differ from those of a broader group of providers. Barriers and promoters relevant to research translation into HAE clinical practice may be different outside of the United States, for example in Europe, where many patients with HAE receive care in rare disease centers from HAE specialists rather than general allergy practitioners. Additionally, insurance prior authorization would not be a barrier in countries in which the public healthcare system does not require this type of review.

c. We amended the Conclusion to acknowledge that our research relates to HAE clinical care in the United States (Manuscript, Line 541-542), as follows (changes are underlined):

This qualitative study revealed a range of barriers to and promoters of research translation into clinical care for patients with HAE in the United States.

**END of RESPONSE TO REVIEWER DOCUMENT**
