# Supplementary material for: Barriers and promoters to adapting research findings to clinical care in hereditary angioedema in the United States: A qualitative study
Source: PLoS One. 2025 Oct 9;20(10):e0329585. doi: 10.1371/journal.pone.0329585 (PMC12510502; doi:10.1371/journal.pone.0329585)
Supplement: Attachment — Submitted filename: Response to Reviewers_082525.docx [file pone.0329585.s003.docx]

**RESPONSE TO REVIEWERS**

**Re: PONE-D-25-13647**

**August 25, 2025**

**To:**	Miquel Vall-Illosera Camps

Senior Staff Editor

PLOS One

Thank you again for your kind consideration of our manuscript (PONE-D-25-13647).

We are grateful for both of the Reviewers’ constructive suggestions.

We have further revised the manuscript in response to Reviewer #2’s suggestions, as described below.

**Comments to the Author**

***1. If the authors have adequately addressed your comments raised in a previous round of review and you feel that this manuscript is now acceptable for publication, you may indicate that here to bypass the “Comments to the Author” section, enter your conflict of interest statement in the “Confidential to Editor” section, and submit your "Accept" recommendation.***

***Reviewer #1****: All comments have been addressed*

***Reviewer #2****: All comments have been addressed*

***2. Is the manuscript technically sound, and do the data support the conclusions?***

***Reviewer #1****: Yes*

***Reviewer #2****: Yes*

***3. Has the statistical analysis been performed appropriately and rigorously?***

***Reviewer #1****: N/A*

***Reviewer #2****: Yes*

4. ***Have the authors made all data underlying the findings in their manuscript fully available?***

***Reviewer #1****: Yes*

***Reviewer #2****: Yes*

***5. Is the manuscript presented in an intelligible fashion and written in standard English?***

***Reviewer #1***: Yes

***Reviewer #2***: Yes

**6. *Review Comments to the Author***

***Reviewer #1****: The AA. have properly edited the manuscript and addressed the critiques raised to the first submitted*

***Reviewer #2****: Dear authors, the novelty reported in your paper is limited.*

*As stated also by another reviewer, the experience of the interviewed physician was low; the majority reported following fewer than five patients. For this reason, the insights reported in the interview are all well known to all the physicians with a larger number of HAE patients. The authors have pointed out in their paper that the field of HAE publication is quite saturated; therefore, I believe it may be worth considering whether this paper adds significant value to the existing body of work.*

**AUTHOR RESPONSE:**

**Little has been previously published about the clinician experience in HAE care**. Most research has focused on the patient experience with HAE. In part because HAE is a rare disease, much less is known about clinician challenges in HAE care compared with a common condition like asthma.

**We therefore do not consider this area of HAE research (clinician challenges) to be saturated**. In fact, we would assert that clinician challenges with HAE need to be more deeply understood before medical care can be improved. Our study, which is novel in its application of qualitative research methods to the topic at hand, provides new information that builds on the findings of previous studies (lines 1031-1042, Track Changes version).

**It is true that clinicians in our study population treated 5 patients with HAE on average annually. However, our data align closely with previous US surveys showing that the majority (70–74%) of US allergy/immunology clinicians treat only 1 to 5 patients with HAE annually**. HAE is a rare disease (estimated prevalence of 1 in 50,000 person). “Physicians with a larger number of HAE patients” practice at HAE subspecialty/referral centers. They would also benefit from knowing more about the specific challenges faced by general allergy practice clinicians like those interviewed in our study.

**To better explain the value of our findings — and how qualitative research can augment the body of knowledge on clinician perspectives — we have made the following changes. (References have been updated accordingly.)**

a. **We clarified how our study fits within the context of previous studies in the Discussion.**

Lines 568-579, Track Changes version:

The results of this study build and expand upon the findings of quantitative survey studies of allergy/immunology health professionals [20, 21]. One study described challenges related to misdiagnosis of HAE, misinterpretation of test results, and other problems with laboratory testing [20]. Additional challenges were lack of clinician and patient knowledge of HAE, patient inability to afford or access treatment, and difficulties incorporating patient preferences into treatment decisions [20]. Regarding long-term HAE prophylaxis, physicians and patients were previously shown to have differing views of treatment burden [21]. Physicians were more likely than patients themselves to view prophylactic medications administered subcutaneously or intravenously as burdensome, inconvenient, or unpleasant. These findings and those of our study highlight the need for enhanced patient-physician communication and shared decision-making about long-term prophylaxis options.

b. **We articulated more clearly the strengths of our study in a new paragraph in the Discussion.**

Lines 609-630, Track Changes version:

One strength of this study is that we uncovered several factors not previously reported in quantitative survey studies of allergy/immunology clinicians that may impact delivery of high-quality HAE patient care [20, 21]. Specifically, we detected clinician difficulties with accessing information on HAE, the use of online chat rooms as an information resource, unfamiliarity with HAE clinical practice guidelines, and lack of use of QoL assessment tools as barriers to research translation. Provision of medication samples and pharmaceutical representative detailing were identified as facilitators of care delivery in our study but not previous quantitative survey studies [20, 21]. In translational health research, qualitative methods are increasingly recognized as valuable ways to reveal stakeholder attitudes, perceptions, and experiences that escape detection using quantitative approaches [23]. Additionally, this study is the first, to our knowledge, to use contemporary qualitative research methods including computer-assisted thematic analysis to examine the experiences of allergy/immunology clinicians related to HAE patient care. Recent qualitative research on HAE has focused on patients rather than clinicians [3, 22, 24,25]. However, the experiences and perspectives of allergy/immunology clinicians, particularly those in general allergy/immunology practices (where most of our respondents worked), also need to be more fully recognized if treatment is to improve for all HAE patients, not just those who receive care in HAE subspecialty centers.

c. **We added this statement about the number of patients with HAE treated annually to the Discussion.**

Lines 640-643, Track Changes version:

Although clinicians in our study reported seeing an average of only 5 patients with HAE annually, this number is consistent with previous surveys of US physicians treating patients with HAE, in which the majority (70–74%) reported treating 1 to 5 patients with HAE in the past year [26].

**7. *Do you want your identity to be public for this peer review*?**

***Reviewer #1***: Yes: Vincenzo Montinaro

***Reviewer #2***: No

**END of RESPONSE TO REVIEWER DOCUMENT**
